# Tumour-associated macrophages: versatile players in the tumour microenvironment

**DOI:** 10.3389/fcell.2023.1261749

**Published:** 2023-10-26

**Authors:** Zoey Zeyuan Ji, Max Kam-Kwan Chan, Alex Siu-Wing Chan, Kam-Tong Leung, Xiaohua Jiang, Ka-Fai To, Yi Wu, Patrick Ming-Kuen Tang

**Affiliations:** ^1^ Department of Anatomical and Cellular Pathology, State Key Laboratory of Translational Oncology, The Chinese University of Hong Kong, Shatin, Hong Kong SAR, China; ^2^ Department of Applied Social Sciences, The Hong Kong Polytechnic University, Kowloon, Hong Kong SAR, China; ^3^ Department of Paediatrics, The Chinese University of Hong Kong, Shatin, Hong Kong SAR, China; ^4^ Key Laboratory for Regenerative Medicine of the Ministry of Education of China, School of Biomedical Sciences, Faculty of Medicine, The Chinese University of Hong Kong, Shatin, Hong Kong SAR, China; ^5^ MOE Key Laboratory of Environment and Genes Related to Diseases, School of Basic Medical Sciences, Xi’an Jiaotong University, Xi’an, China

**Keywords:** tumour-associated macrophages, tumour microenvironment, immunotherapy, macrophage plasticity, macrophage-myofibroblast transition, macrophage-neuron transition

## Abstract

Tumour-Associated Macrophages (TAMs) are one of the pivotal components of the tumour microenvironment. Their roles in the cancer immunity are complicated, both pro-tumour and anti-cancer activities are reported, including not only angiogenesis, extracellular matrix remodeling, immunosuppression, drug resistance but also phagocytosis and tumour regression. Interestingly, TAMs are highly dynamic and versatile in solid tumours. They show anti-cancer or pro-tumour activities, and interplay between the tumour microenvironment and cancer stem cells and under specific conditions. In addition to the classic M1/M2 phenotypes, a number of novel dedifferentiation phenomena of TAMs are discovered due to the advanced single-cell technology, e.g., macrophage-myofibroblast transition (MMT) and macrophage-neuron transition (MNT). More importantly, emerging information demonstrated the potential of TAMs on cancer immunotherapy, suggesting by the therapeutic efficiency of the checkpoint inhibitors and chimeric antigen receptor engineered cells based on macrophages. Here, we summarized the latest discoveries of TAMs from basic and translational research and discussed their clinical relevance and therapeutic potential for solid cancers.

## Introduction

Tumour microenvironment (TME) is crucial for cancer initiation, progression, and drug resistance. TME is formed by various fundamental constituents including stromal cells and immune cells (Cassetta et al., 2019; Li et al., 2023; Wang et al., 2023). Cancer development can be facilitated by tissue inflammation (Nost et al., 2021; Rajamaki et al., 2021). Despite the diverse inflammatory components in various cancer types (Cheng et al., 2021), increasing evidence demonstrated the importance of macrophages in the progression of solid cancers (Christofides et al., 2022). Macrophage is the key inflammatory effector cells, better understanding its roles may uncover effective therapeutic strategy for cancer (Coussens et al., 2013).

Interestingly, macrophages are versatile in tissues under inflammation including cancer (Maier et al., 2020; Vayrynen et al., 2021; Xue et al., 2021; Nalio Ramos et al., 2022). Their phenotypes and functions are broadly categorized into pro-inflammatory M1 and anti-inflammatory M2 (Cho et al., 2022; Zhou et al., 2022). M1 macrophages eliminate cancer cells by phagocytosis, antibody-dependent cytotoxicity, vascular damage, and tumour necrosis. M2 macrophages promote tumour growth and progression via enhancing cancer cell survival, angiogenesis and immune suppression (Zhao et al., 2020; Chen et al., 2021; Ren et al., 2022). Beyond M1/M2 polarization, new transition mechanisms for TAMs have been recently identified by single-cell bioinformatic studies including MMT (Tang et al., 2022a) and MNT (Tang et al., 2022b), their roles in cancer remain unclear.

Clinical studies highlight the crucial roles of macrophages in cancer therapy response and resistance, including chemotherapy, radiotherapy, and PDL1-based immunotherapy (Furuse et al., 2020; Liu et al., 2020). Moreover, clinical trials of macrophage-targeted therapies have been started such as the engineered mononuclear phagocytes (Brempelis et al., 2020) and chimeric antigen receptor macrophages (CAR-M) (Klichinsky et al., 2020; Wang et al., 2022), these therapeutic approaches stem from bench-top discoveries like recruitment and differentiation (Hannan et al., 2023), functional reprogramming (Willingham et al., 2012), and integration (Dang et al., 2021), highlighting the importance of basic research and preclinical study for the development of effective cancer treatment.

In this review, we systematically summarized the functional roles and underlying mechanisms of macrophages in TME for cancer formation and progression, their translational potential, and related studies on patients for overcoming the barriers of conventional cancer treatments as well as the latest immunotherapy resistance in the clinic. Finally, we also discussed the prospects and further directions of TAMs in the clinical development for cancer treatment.

## Physiological roles of macrophages

Macrophages release cytokines and chemokines for recruiting immune cells for wound healing and blood vessel formation (Hernandez et al., 2022), including vascular endothelial growth factor (VEGF) (Lu et al., 2020) and transforming growth factor-beta (TGF-β) (Chung et al., 2018). Macrophages maintain tissue integrity (Mosser et al., 2021), clearing apoptotic cells (Dooling et al., 2023), debris (Kim et al., 2020), and pathogens (Nau et al., 2002) via cell-mediated phagocytosis, where the targets are recognized by pattern recognition receptors (PRRs) dependent mechanisms (Li and Wu, 2021) i.e., Toll-like receptors (TLRs) (Irizarry-Caro et al., 2020) and NOD-like receptors (NLRs) (Fekete et al., 2018; Frising et al., 2022).

Furthermore, macrophages are involved in innate and adaptive immune responses by recognizing pathogen-associated molecular patterns (PAMPs) (Greene et al., 2022) and damage-associated molecular patterns (DAMPs) (Serbulea et al., 2018; Neu et al., 2022) through PRRs. Activated macrophages produce pro-inflammatory cytokines, i.e., tumour necrosis factor-alpha (TNF-α) (Lee et al., 2021; Lechner et al., 2022; Tanito et al., 2023) and interleukin-12 (IL-12) (Luo et al., 2022; Pfirschke et al., 2022), to promote inflammation and activate other immune cells. Macrophages also process and present antigens to T cells via major histocompatibility complex (MHC) molecules aiding adaptive immune response (Mascarau et al., 2023; van Elsas et al., 2023). Interestingly, tissue-specific macrophages display unique functions. For example, alveolar macrophages in lung, express high levels of surfactant protein A (SP-A) (Bain and MacDonald, 2022; Garcia-Fojeda et al., 2022; Yau et al., 2023) and surfactant protein D (SP-D) receptors (Guo et al., 2019; Hsieh et al., 2023) for clearing inhaled particles and pathogens. Liver-resident macrophages, Kupffer cells, express various scavenger receptors (Taban et al., 2022), complement receptors (Wen et al., 2021), and Fc receptors (Pfefferle et al., 2023), filtering blood-borne pathogens (Zhao et al., 2022a), toxins (Kermanizadeh et al., 2019), and debris (Liu and Sun, 2023).

**FIGURE 1:**
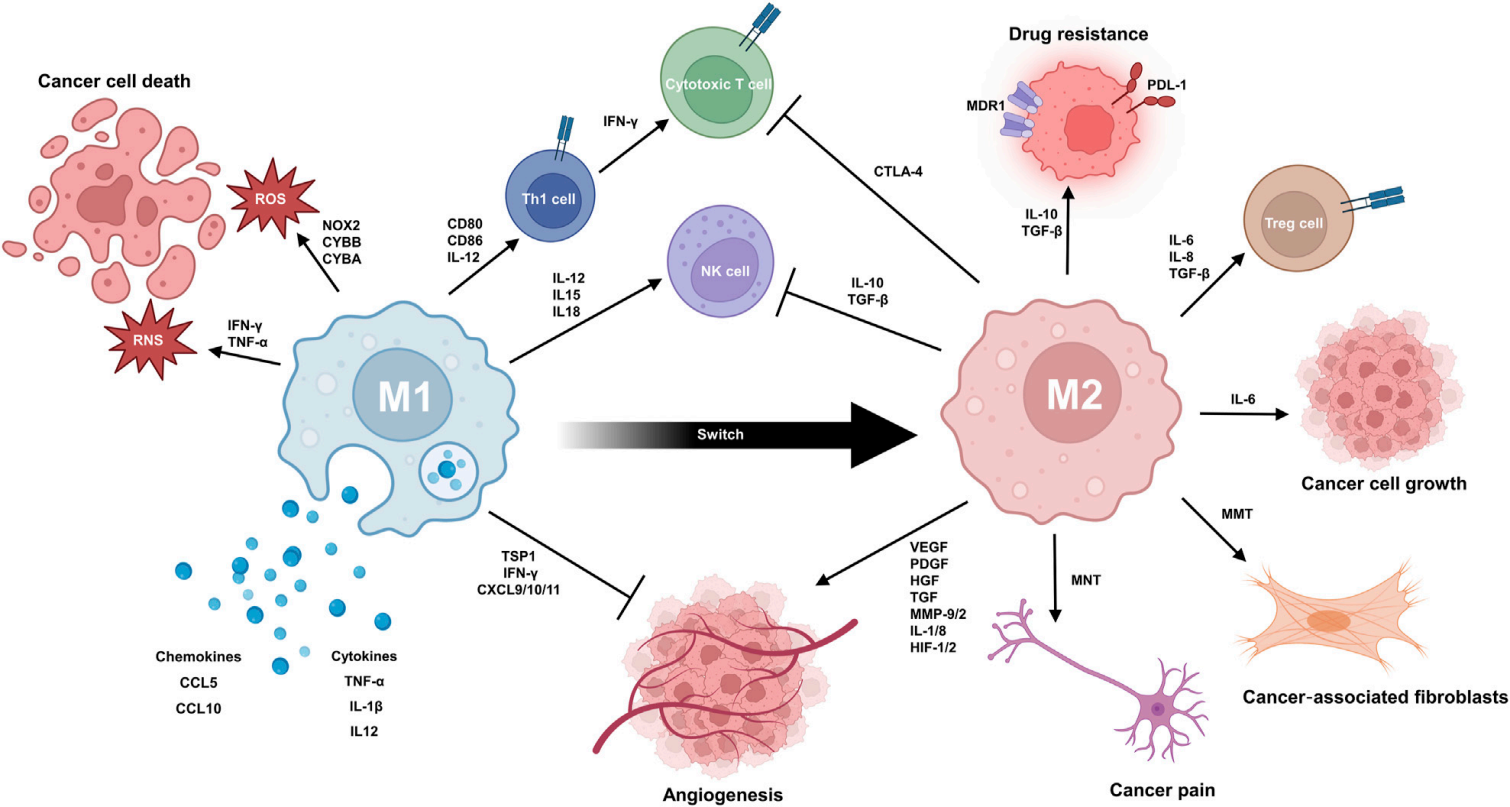
TAMs play a complex dual role in the progression of cancer. M1 TAMs contribute to the anticancer response via multiple mechanisms. They can produce reactive oxygen species (ROS) and reactive nitrogen species (RNS) to cause oxidative damage and kill cancer cells. The secretion of pro-inflammatory cytokines and chemokines (e.g., TNF-α, IL1B, IL12A/B, CCL5, and CXCL10) can mobilize other anticancer immune cells, like T cells and NK cells, into the TME. Anti-angiogenesis is promoted by secretion of thrombospondin-1 and angiostatic chemokines like CXCL9, CXCL10, and CXCL11. TAMs also express MHC class I and II molecules for antigen presentation to further priming and activation of T cells. The interaction between CD80/CD86 on TAMs and CD28 on T cells provides a second signal for T cell activation. M2 TAMs promote immunosuppression, angiogenesis, and tumour growth/metastasis while contributing to drug resistance. Immunosuppression involves secretion of TGF-β and IL-10, expression of PD-L1, and CCL22-induced Treg activation. In angiogenesis, TAMs secrete factors like VEGF, FGFs, PDGF, HGF, MMPs, and IL-8/1. During tumour growth and metastasis, M2 TAMs enhance proliferation, migration, and invasion. Factors like EGF, PDGF, VEGF, CCL-10, and MMPs play key roles. TAM can also undergo transformation to MNT and MMT, resulting in the generation of cancer pain and cancer-associated fibroblast. In drug resistance, TAM-derived TGF-β, IL-6/8, and PDGF stimulate survival pathways and enhance DNA repair in cancer cells. It is noteworthy that macrophages can switch from M1 phenotype to M2 phenotype during tissue repair.

Macrophages are classified into M1 and M2 phenotypes (Guilliams and Svedberg, 2021; De Vlaminck et al., 2022). M1 macrophages express high level of pro-inflammatory cytokines like Interleukin-1β (IL-1β), Interleukin-6 (IL-6), IL-12, Interleukin-23 (IL-23), and TNF-α (Hou et al., 2018; Akhtari et al., 2021; Beyranvand Nejad et al., 2021; Gunassekaran et al., 2021) polarized by Th1 cytokines including GM-CSF, TNF-α, and interferon-gamma (IFN-γ) (Wu et al., 2022a; Zhao et al., 2022b; Cho et al., 2022; Zhang et al., 2023), whereas, M2 macrophages actively produce anti-inflammatory cytokines Interleukin-10 (IL-10) and TGF-β (Nagata et al., 2019; Yang et al., 2023a) and polarized by Th2 cytokines like Interleukin-4 (IL-4) and Interleukin-13 (IL-13) (Celik et al., 2020; Lundahl et al., 2022). For metabolism, M1 macrophages rely on glycolysis (Yu et al., 2020; Mouton et al., 2023), while M2 macrophages depend on oxidative phosphorylation (Xu et al., 2021a; Zhou et al., 2022). During tissue repair, macrophages switch from an M1-like to an M2-like phenotype (Kim et al., 2019a; Alhamdi et al., 2019; Kohno et al., 2021). Interestingly, M1/M2 homeostasis is disrupted by inhibition of aspartate-aminotransferase (Wu et al., 2020a) and N-glycosylation (Wu et al., 2020a; Hu et al., 2023), altering immune responses and tissue damage. Moreover, various polarization and activation markers coexist in tissues, and factors like the macrophage-inducible C-type lectin (MINCLE) (Maier et al., 2020; Xue et al., 2021) or TLRs (Vidyarthi et al., 2018; Zhou et al., 2022) impact their balance. TAMs play multifaceted roles in cancer progression that are both beneficial and detrimental, highlighting the dual nature of their involvement (Figure 1).

## Anticancer effects of TAMs

### Reactive species production

M1 TAMs produce reactive oxygen species (ROS), mediated by NADPH oxidase (Fang et al., 2022; Tlili et al., 2023), causing cancer cell death. Activation by IFN-γ and TNF-α prompts TAMs to generate reactive nitrogen species (RNS) via nitric oxide synthase (iNOS) (Zhang et al., 2021a; Wei et al., 2022). Collectively, these ROS and RNS induce oxidative damage on cancer cells, leading to direct cancer cell-killing effect (Liang et al., 2019; Huang et al., 2022; Qi et al., 2022; Kidwell et al., 2023).

### Pro-inflammatory cytokine and chemokine

TAMs secrete pro-inflammatory cytokines for mobilizing anticancer cells (e.g., T cells and natural killer cells) into TME, including TNF-α (Jiang et al., 2019; Kaplanov et al., 2019; Tu et al., 2021a), IL1B (interleukin-1 beta) (Revu et al., 2018), IL12A and IL12B (subunits of IL-12) (Yen et al., 2022). TAMs also produce chemokines, e.g., C-C Motif Chemokine Ligand 5 (CCL5) and C-X-C motif chemokine ligand 10 (CXCL10) to recruit and activate other immune cells to TME, driven by pro-inflammatory transcription factor NF-κB (nuclear factor kappa-light-chain-enhancer of activated B cells) (Taki et al., 2018). Furthermore, M1 macrophages produce IL-12, prompting CD4^+^ T cells towards Th1 phenotype (Zhao et al., 2022b), these Th1 cells will produce IFN-γ to activate cytotoxic CD8^+^ T cells in TME (Greaney et al., 2020; Liu et al., 2022). M1 macrophages also stimulate NK cell activation by IL-12, IL-15 and IL-18 (Mattiola et al., 2015).

### Anti-angiogenesis

M1 macrophages secrete angiostatic factor thrombospondin-1(TSP1) (Yang et al., 2019; Kumar et al., 2020) for inhibiting angiogenesis by interacting with an endothelial cell receptor CD36 in various cancers, including hepatocellular carcinoma (Aburima et al., 2021). Moreover, M1 macrophages produce additional angiostatic chemokines to block vessel formation via CXCR3 (C-X-C Motif Chemokine Receptor 3) dependent mechanism, including CXCL9, 10, 11 (C-X-C Motif Chemokine Ligand 9, 10, 11) (Romagnani et al., 2004; Sahraei et al., 2019).

### Antigen presentation

M1 macrophages express MHC class I and II molecules (Haloul et al., 2019; Ahmed and Ismail, 2020) to present cancer antigens, involving several genes, including MHC class I (Yao et al., 2020; Desterke et al., 2021; Piatakova et al., 2021) and II (He et al., 2021; Tang et al., 2022c; Scavuzzi et al., 2022). The interaction of MHC molecules with T cell receptors amplifies anti-tumour host immune response (Guerriero, 2019; Kawasaki et al., 2022). Interaction between CD80 and CD86 on the M1 macrophage and CD28 on the T cell also provides crucial second signal for T cell activation (Trzupek et al., 2020).

## Pro-tumour effects of TAM

### Immunosuppression

TAMs contribute to immunosuppression in TME, including lung adenocarcinoma (LUAD) and bladder cancer (BLCA). They inhibit the anticancer activities of NK cells primarily through producing TGF-β (Nunez et al., 2018) and IL-10 (Xu et al., 2022). TGF-β hampers NK cell cytotoxicity by downregulating NKG2D receptor expression (Lazarova and Steinle, 2019). IL-10 inhibits the production of the anticancer cytokine IFN-γ in NK cells (Wang et al., 2021a). TAMs in these diverse cancer types express programmed death-ligand 1 (PD-L1) (Sumitomo et al., 2019; Shinchi et al., 2022; Xia et al., 2022; Elomaa et al., 2023), which interacts with the PD-1 receptor on T cells (Pereira et al., 2023; Puig-Saus et al., 2023) and NK cells (Zhou et al., 2023a; van der Sluis et al., 2023), leading to their exhaustion and promoting tumour immune evasion. TAM-derived CCL22 (C-C Motif Chemokine Ligand 22) contributes to the recruitment and activation of regulatory T cells (Tregs) (Rapp et al., 2019; Chen et al., 2022a), inducing immunosuppression in TME (Kraaij et al., 2010; Erlandsson et al., 2019). TAMs also enhance immunosuppressive function of Tregs, promote the transition of conventional CD4^+^ T cells into Tregs (Morhardt et al., 2019; Saraiva et al., 2020; Maldonado et al., 2022), and activate myeloid-derived suppressor cells (MDSCs) via IL-10 (Yu et al., 2018; Yogev et al., 2022) and TGF-β (Becker et al., 2018; Astarita et al., 2023). Furthermore, TAMs express immune checkpoint molecule cytotoxic T-lymphocyte-associated protein 4 (CTLA-4) (Guan et al., 2021), interacting with CD80/CD86 of Tregs to amplify their immunosuppressive effects (Zappasodi et al., 2021; Kennedy et al., 2022).

### Angiogenesis

TAMs play pivotal role in augmenting angiogenesis within the TME, integral to cancer progression (Cheng et al., 2021). Essential for tumor growth and metastasis (Liu et al., 2023a; Natale and Bocci, 2023), angiogenesis provides TME with necessary nutrients and oxygen, aiding in the growth of cancer cells (Schaaf et al., 2018; Lugano et al., 2020; Schito and Rey, 2020). TAMs secret factors for promoting angiogenesis, including VEGF (Schaaf et al., 2018), fibroblast growth factors (FGF1 and FGF2) (Schaaf et al., 2018; Im et al., 2020), platelet-derived growth factor (PDGF) (Ntokou et al., 2021), hepatocyte growth factor (HGF) (Choi et al., 2019; Dong et al., 2019), matrix metalloproteinases (MMP-9, MMP-2) (Diwanji and Bergmann, 2020; Tian et al., 2022), and cytokines like IL-8 and IL-1 (Liu et al., 2023b; Yang et al., 2023b). VEGF is crucial for tumoural angiogenesis (Lai et al., 2019; Hwang et al., 2020). Moreover, TAMs are concentrated in the hypoxic zones of tumours (Bai et al., 2022), where they upregulate the expression of numerous angiogenic genes including Hypoxia-inducible factors (HIF)-1 and −2 (Jeong et al., 2019; Cowman et al., 2020) for enhancing the production of angiogenic factors like VEGF in TME (Roda et al., 2012).

### Cancer growth and metastasis

M2 TAMs promote primary tumour development and metastasis (Yao et al., 2018; Li et al., 2019a; Tu et al., 2021b). They increase tumour proliferation in breast cancer (Chen et al., 2022b; Zhou et al., 2023b), endometrial cancer (Xiao et al., 2020; Gu et al., 2021), and renal cell carcinoma (Xie et al., 2021; Ishii et al., 2022). Furthermore, M2 TAMs secrete Epidermal Growth Factor (EGF) (Zeng et al., 2019; Wu et al., 2020b), which binds to EGFR on cancer cells, for activating their growth signaling including MAPK/ERK (Liang et al., 2022) and PI3K/Akt pathways (Zhang et al., 2021b), promoting cell motility and invasion (Haque et al., 2019; Zeng et al., 2019; Onal et al., 2021). Growth Factor PDGF (Turrell et al., 2023) secreted from TAMs also contributes to tumour cell proliferation. Tumour metastasis is defining characteristic of advanced cancer stage, TAM-derived EGF accelerates metastasis by activating the EGFR-ERK signaling and inhibiting the expression of lncRNA LIMT (Zeng et al., 2019) in the epithelial ovarian cancer.

At the pre-metastasis stage, TAMs secrete VEGF, CCL-10 and MMPs, which remodel distant tissues to create pre-metastatic niche (Kim et al., 2019b; Winkler et al., 2020). TAMs release inflammatory factors TNF-α, IL-6, and IL-11 (Kaplanov et al., 2019; Yu et al., 2019; Beyranvand Nejad et al., 2021) to enhance cancer cell survival and proliferation by activating NF-κB and STAT3 pathways (Dorrington and Fraser, 2019; Balic et al., 2020). TGF-β from TAMs activates TGF receptors on cancer cells, initiating SMAD signaling for their growth (Chung et al., 2023; Lv et al., 2023). Importantly, TAM-derived TGF-β induces epithelial-to-mesenchymal transition (EMT) of cancer cells (Cai et al., 2019; Tiwari et al., 2021), allowing them to migrate into surrounding tissue and vasculature (Dongre and Weinberg, 2019; Wang et al., 2021b). Additionally, TAMs-secreted MMPs, such as MMP2 and MMP9 (Wang and Khalil, 2018; Liu et al., 2019; Muniz-Bongers et al., 2021), degrade the ECM in TME (Marigo et al., 2020), enabling metastasis into the bloodstream or lymphatic system (Winkler et al., 2020). TAMs produce chemokines like CCL18 and CCL22 (She et al., 2018; Kimura et al., 2019; Zhou et al., 2019; Chen et al., 2022a) to promote tumour cell migration. TAMs also release proteases like cathepsins (CTSB, CTSD) (Loeuillard et al., 2020; Shi et al., 2022) to stimulate tumour cells to produce tissue inhibitors of metalloproteinases, enhancing ECM degradation and metastasis (Bissinger et al., 2021).

TAMs transformation also contributes to cancer progression. Besides M1/M2 polarization, single-cell RNA-sequencing revealed new TAM phenomena. Macrophage to MNT, a process where TAMs transform into neuron-like cells contributing to the formation of cancer pain (Tang et al., 2022b). MMT, where TAMs trans-differentiate into myofibroblasts for increasing abundance of pro-tumour cancer-associated fibroblasts (CAFs) in TME, enhancing the progression of non-small-cell lung carcinoma (NSCLC) (Tang et al., 2022a).

### Drug resistance

TAMs are associated with resistance of cancer therapy (Mantovani et al., 2022). TAM-derived TGF-β upregulates the expression of multidrug resistance protein 1 (MDR1) in cancer cells (Badmann et al., 2020), leading to drug resistance. TAMs secrete IL-6 and IL-8 (Ahmed et al., 2021; Radharani et al., 2022), associated with resistance to therapies including EGFR tyrosine kinase inhibitors. TAMs-secreted PDGF enhances DNA repair in cancer cells against radiation therapy (Sakama et al., 2021).

### Interplay between TME and cancer stem cells

The dynamic relationship between the TME and cancer stem cells (CSCs) is central to understanding the roles of TAMs. CSCs, distinguished by their pronounced expression of stemness markers like SOX2, NANOG, and OCT4 (Zhou et al., 2021), actively drive self-renewal, differentiation, and are influenced by signals from TME (Yang et al., 2020). Key pathways such as TGF-β, Wnt, and Hedgehog (Li et al., 2019b; Zhu et al., 2019; Wu et al., 2022b) mold the genetic landscape of CSCs. The crosstalk between CSCs and TME involves factors including IL-6 (Orange et al., 2023), IL-8 (Sun et al., 2018), IL-1β (Eyre et al., 2019), MMPs (Jin and Jin, 2020), VEGF (Lopez de Andres et al., 2020), and TGF-β1 (Yuan et al., 2022), which are encapsulated within extracellular vehicles (EVs) (Su et al., 2021; Cao et al., 2022). Given the immunomodulatory role of CSCs, further studies are essential to understand the clinical implications.

Importantly, interaction between TAMs and CSCs fosters an immunosuppressive TME (Wu et al., 2023). CSCs promote macrophage recruitment and polarization by ILs, ECM, TGF-β, and periostin (Ning et al., 2018; Kesh et al., 2020; Taniguchi et al., 2020; Li et al., 2022a; Lin et al., 2022). Moreover, TAMs increase CD47 expression in pancreatic, liver and lung cancer stem cells (Cioffi et al., 2015; Liu et al., 2017; Ruiz-Blazquez et al., 2021). When linked to SIRPα on macrophages, CD47 expression protects CSCs against immune cell-mediated phagocytosis (Li et al., 2018). TAM-secreted factors also upregulate immunological checkpoints like PD-L1 (Muraoka et al., 2019; Pu and Ji, 2022). The intricate interplay between CSCs and TAMs creates immunosuppressive TME, enhancing the survival of CSC and hindering tumour eradication post-immunotherapy.

## Macrophage-targeted antitumour therapy

TAMs are essential for cancer immunotherapy (Lin et al., 2019). Macrophage-targeted treatments often deplete macrophages, modify their phenotypes, or enhance antigen presentation activity of TAM (Cassetta and Pollard, 2018). Combined with chemotherapy, radiation, or immunotherapy, these techniques may increase host antitumor immunity. They have been studied in animal models and clinical studies with immunological checkpoints and other immunotherapies (Table 1).

**TABLE 1:** Selected clinical trials of drugs targeting TAMs.

Compound	Clinical phase	Tumour type	Status	NCT identifier	Year
CSF1R inhibitors					
PLX3397	Phase1	Drug Interaction Potential	Completed	NCT03291288	2017
	Phase3	Tenosynovial Giant Cell Tumour	Active_Not_Recruiting	NCT04488822	2020
	Phase4	Tenosynovial Giant Cell Tumour	Active_Not_Recruiting	NCT04526704	2020
	Phase2	Tenosynovial Giant Cell Tumour	Recruiting	NCT04703322	2021
HMPL-012	Phase2	Advanced Solid Tumours	Completed	NCT04169672	2019
	Phase2	Thyroid Cancer	Unknown	NCT04524884	2020
	Phase2	Neuroendocrine Tumours	Active_Not_Recruiting	NCT04579679	2020
	Phase2	Advanced Colorectal Cancer	Not_Yet_Recruiting	NCT04734249	2021
	Phase2	Advanced Colorectal Cancer	Recruiting	NCT04764006	2021
	Phase2	Advanced Non-Small Cell Lung Cancer	Recruiting	NCT04922658	2021
	Phase1 and 2	Advanced Colorectal Cancer	Recruiting	NCT04929652	2021
	Phase1	Small Cell Lung Carcinoma	Recruiting	NCT04996771	2021
	Phase2	Carcinoma, Non-Small-Cell Lung	Recruiting	NCT05003037	2021
	Phase2	Refractory Metastatic Digestive System Carcinoma and Peritoneal Cancer	Recruiting	NCT05030246	2021
	Na	Biliary Tract Cancer	Recruiting	NCT05056116	2021
	Phase1	Neuroendocrine Tumours and Non-hematologic Malignancy	Recruiting	NCT05077384	2021
	Phase1 and 2	Solid Tumour	Active_Not_Recruiting	NCT05093322	2021
	Phase2	Neuroendocrine Neoplasm	Recruiting	NCT05165407	2021
	Phase2	Hepatocellular Carcinoma	Recruiting	NCT05171439	2021
	Phase2	Breast Cancer and Breast Cancer Female	Recruiting	NCT05186545	2022
	Phase1 and 2	Pancreatic Cancer	Recruiting	NCT05218889	2022
	Phase2	Gastric Adenocarcinoma	Not_Yet_Recruiting	NCT05235906	2022
	Phase2	Pancreatic Neoplasms	Not_Yet_Recruiting	NCT05481463	2022
	Phase2	Pancreatic Neoplasms	Not_Yet_Recruiting	NCT05481476	2022
	Phase2	Advanced Solid Tumours	Not_Yet_Recruiting	NCT05527821	2022
	Phase2	Small Cell Lung Cancer	Not_Yet_Recruiting	NCT05595889	2022
	Phase2	Pancreatic Carcinoma	Recruiting	NCT05627427	2022
	Phase2	Extensive-stage Small-cell Lung Cancer	Not_Yet_Recruiting	NCT05668767	2022
	Phase1 and 2	Metastatic Triple-negative Breast Cancer	Not_Yet_Recruiting	NCT05746728	2023
	Phase1 and 2	Unresectable Locally Advanced	Not_Yet_Recruiting	NCT05832892	2023
	Phase1 and 2	Small Cell Lung Cancer	Not_Yet_Recruiting	NCT05882630	2023
	Phase2	Pancreatic Cancer	Recruiting	NCT05908747	2023
DCC-3014	Phase1	Advanced Sarcoma cancer	Active_Not_Recruiting	NCT04242238	2020
	Phase3	Giant Cell Tumour	Active_Not_Recruiting	NCT05059262	2021
	Phase1 and 2	Advanced Malignant Neoplasm	Recruiting	NCT03069469	2017
CS2164	Phase1	Small Cell Lung Cancer	Recruiting	NCT03216343	2017
	Phase1 and 2	Ovarian Cancer	Completed	NCT03166891	2017
	Phase2	Ovarian Cancer	Completed	NCT03901118	2019
	Phase3	Small Cell Lung Cancer	Recruiting	NCT04830813	2021
	Phase3	Ovarian Cancer and Relapsed or Refractory and Chiauranib and Paclitaxel	Recruiting	NCT04921527	2021
	Phase1 and 2	Small-cell Lung Cancer and Advanced Solid Malignant Tumour	Recruiting	NCT05271292	2022
Q702	Phase1	Solid Tumour and Advanced Cancer and Metastatic Cancer	Recruiting	NCT04648254	2020
	Phase1 and 2	Esophageal Cancer, Gastric Cancer, Hepatocellular Cancer and Cervical Cancer	Recruiting	NCT05438420	2022
TPX-0022	Phase1 and 2	Advanced Solid Tumour	Active_Not_Recruiting	NCT03993873	2019
X-82	Phase1	Solid Tumour	Terminated	NCT03511222	2018
	Phase1 and 2	Thymic Carcinoma, Non-small Cell Lung Cancer and Small-Cell Lung Cancer	Active_Not_Recruiting	NCT03583086	2018
	Phase1	Advanced Malignant Solid Tumours	Active_Not_Recruiting	NCT03792958	2019
	Phase2	Extensive-stage Small Cell Lung Cancer	Active_Not_Recruiting	NCT04373369	2020
Chemokine inhibitors					
BMS-813160	Phase1 and 2	Colorectal Cancer and Pancreatic Cancer	Active_Not_Recruiting	NCT03184870	2017
	Phase1 and 2	Pancreatic Ductal Adenocarcinoma	Active_Not_Recruiting	NCT03496662	2018
	Phase1 and 2	Locally Advanced Pancreatic Ductal Adenocarcinoma	Recruiting	NCT03767582	2018
	Phase2	Non-small Cell Lung Cancer and Hepatocellular Carcinoma	Recruiting	NCT04123379	2019
Maraviroc	Phase1	Metastatic Colorectal Cancer and MSS	Completed	NCT03274804	2017
	Phase1	Colorectal Cancer Metastatic and Pancreatic Cancer Metastatic	Unknown	NCT04721301	2021
	Phase1 and 2	HIV and Hematologic Malignancies	Recruiting	NCT05470491	2022
Anti-CD47/SIRPα antibodies					
Hu5F9-G4	Phase1	Hematological Malignancies	Active_Not_Recruiting	NCT03248479	2017
	Phase1	Ovarian Cancer	Completed	NCT03558139	2018
	Phase1	Acute Myeloid Leukemia	Terminated	NCT03922477	2019
	Phase1 and 2	Mycosis Fungoides and	Recruiting	NCT04541017	2020
	Phase1	Follicular Lymphoma	Recruiting	NCT04599634	2020
	Phase1	High Risk Neuroblastoma, Recurrent Neuroblastoma and Resectable Osteosarcoma	Suspended	NCT04751383	2021
	Phase2	Myeloid Malignancies	Active_Not_Recruiting	NCT04778410	2021
	Phase2	Solid Tumour	Recruiting	NCT04827576	2021
	Phase2	Triple-Negative Breast Cancer	Recruiting	NCT04958785	2021
	Phase1	Brain Cancer	Recruiting	NCT05169944	2021
	Phase2	Metastatic Colorectal Cancer	Recruiting	NCT05330429	2022
	Phase1	Advanced Malignant Solid Neoplasm	Not_Yet_Recruiting	NCT05807126	2023
BI 754091	Phase1	Neoplasms and Carcinoma, Non-Small-Cell Lung	Completed	NCT03156114	2017
	Phase1	Neoplasms and Neoplasm Metastasis and Carcinoma, Non-Small-Cell Lung	Terminated	NCT03166631	2017
	Early_Phase1	Neoplasms	Active_Not_Recruiting	NCT03433898	2018
	Phase1	Non-squamous, Non-Small-Cell Lung Cancer and Neoplasms	Active_Not_Recruiting	NCT03468426	2018
	Phase2	Neoplasm Metastasis	Active_Not_Recruiting	NCT03697304	2018
	Phase1	Carcinoma, Non-Small-Cell Lung and Head and Neck Neoplasms	Terminated	NCT03780725	2018
	Phase1	Neoplasms	Recruiting	NCT03964233	2019
	Phase1	Neoplasms	Completed	NCT03972150	2019
	Phase1	Solid Tumour, Adult	Recruiting	NCT03990233	2019
	Phase1 and 2	Colorectal Cancer	Recruiting	NCT04046445	2019
	Phase1	Neoplasm	Completed	NCT04138823	2019
	Phase1	Neoplasms	Active_Not_Recruiting	NCT04147234	2019
	Phase2	Anal Canal Squamous Cell Carcinoma	Withdrawn	NCT04499352	2020
	Phase1	Solid Tumours	Completed	NCT04653142	2020
	Phase2	Squamous Cell Carcinoma	Recruiting	NCT04719988	2021
	Phase1	Colorectal Neoplasms, Carcinoma and Non-Small-Cell Lung	Recruiting	NCT04752215	2021
	Phase1	Neoplasms	Recruiting	NCT04958239	2021
	Phase1	Head and Neck Squamous Cell Carcinoma	Recruiting	NCT05249426	2022
	Phase1	Solid Tumours	Recruiting	NCT05471856	2022
ALX148	Phase1	Metastatic Cancer and Solid Tumour and Advanced Cancer and NonHodgkin Lymphoma	Active	NCT03013218	2017
	Phase2 and 3	Gastric Cancer	Recruiting	NCT05002127	2021
	Phase1 and 2	HER2-expressing Cancers	Recruiting	NCT05027139	2021
	Phase2	Microsatellite Stable Metastatic Colorectal Cancer	Recruiting	NCT05167409	2021
	Phase2	Ovarian Cancer	Recruiting	NCT05467670	2022
	Phase2	Oropharynx Cancer	Not_Yet_Recruiting	NCT05787639	2023
	Phase1	HER2-positive Breast Cancer and Metastatic Cancer	Recruiting	NCT05868226	2023
AO-176	Phase1 and 2	Solid Tumour	Active_Not_Recruiting	NCT03834948	2019
IBI188	Phase1	Advanced Malignancies	Completed	NCT03763149	2018
SRF231	Phase1	Advanced Solid Cancers and Hematologic Cancers	Completed	NCT03512340	2018
Agonist anti-CD40 antibodies					
SEA-CD40	Phase2	Melanoma and Carcinoma, Non-Small- Cell Lung	Active_Not_Recruiting	NCT04993677	2021
APX005M	Phase1 and 2	Solid Cancers	Completed	NCT03123783	2017
	Phase2	Esophageal Cancer, Gastric Cancer and Hepatocellular Cancer	Active_Not_Recruiting	NCT03165994	2017
	Phase1	Glioblastoma Multiforme, Nos and Ependymoma, NOS and Medulloblastoma	Active_Not_Recruiting	NCT03389802	2018
	Phase1	Advanced Melanoma, Non-small Cell Lung Cancer and Renal Cell Carcinoma	Active_Not_Recruiting	NCT03502330	2018
	Phase1	Metastatic Melanoma	Terminated	NCT03597282	2018
	Phase2	Soft Tissue Sarcoma	Recruiting	NCT03719430	2018
	Phase2	Locally Advanced Rectal Adenocarcinoma	Active_Not_Recruiting	NCT04130854	2019
	Phase2	Ovarian Cancer	Not_Yet_Recruiting	NCT05201001	2022
	Phase1 and 2	Pancreatic Cancer	Recruiting	NCT05419479	2022
CDX-1140	Phase1	Solid Tumours	Completed	NCT03329950	2017
	Phase1 and 2	Non-Small Cell Lung Cancer	Recruiting	NCT04491084	2020
	Phase1	Malignant Epithelial Neoplasms	Recruiting	NCT04520711	2020
	Phase2	Pancreatic Cancer	Recruiting	NCT04536077	2020
	Phase1	Breast Cancer and Melanoma	Recruiting	NCT04616248	2020
	Phase1	Metastatic Triple Negative Breast Cancer	Recruiting	NCT05029999	2021
	Phase2	Solid Tumours	Not_Yet_Recruiting	NCT05231122	2022
	Phase1	Malignant Epithelial Neoplasms	Enrolling_By_Invitation	NCT05349890	2022
NG-350A	Phase1	Metastatic Cancer and Epithelial Tumour	Completed	NCT03852511	2019
	Phase1	Epithelial Tumour and Metastatic Cancer	Recruiting	NCT05165433	2021
TLR agonists					
Imiquimod	Phase1	Carcinoma, Non-Small-Cell Lung Cancer	Unknown	NCT03057340	2017
	Early_Phase1	Cervical Intraepithelial Neoplasia	Active_Not_Recruiting	NCT03196180	2017
	NA	Cervical Intraepithelial Neoplasia 3	Unknown	NCT03206138	2017
	Phase2	High Grade Intraepithelial Neoplasiaand Cervix Cancer	Completed	NCT03233412	2017
	Phase2	Basal Cell Carcinoma, Basal Cell Carcinoma of Skin and Invasive Carcinoma	Recruiting	NCT03534947	2018
	Phase1 and 2	Primary/Relapsed Acute Lymphoblastic Leukemia (ALL) of Childhood, Adolescents and Young Adults	Unknown	NCT03559413	2018
	Phase1	Solid Tumours	Recruiting	NCT03872947	2019
	Phase1	Malignant Glioma	Recruiting	NCT03893903	2019
	Phase1	Metastatic Breast Cancer	Terminated	NCT03982004	2019
	Phase1	Melanoma	Unknown	NCT04072900	2019
	Early_Phase1	Basal Cell Carcinoma	Completed	NCT04279535	2020
	Phase1	Glioblastoma	Active_Not_Recruiting	NCT04642937	2020
	Early_Phase1	Oral Cancer	Recruiting	NCT04883645	2021
	Phase1	Bladder Cancer and Bladder	Recruiting	NCT05055050	2021
	Phase3	Basal Cell Carcinoma	Not_Yet_Recruiting	NCT05212246	2022
	Phase1	Bladder Cance	Recruiting	NCT05375903	2022
Resiquimod	Phase1	Tumours	Completed	NCT00821652	2009
	Phase1 and 2	Advanced Malignancies	Completed	NCT00948961	2009
	Phase2	Melanoma	Completed	NCT00960752	2009
	Phase2	Bladder Cancer	Terminated	NCT01094496	2010
	Phase2	Glioma and Glioblastoma	Active_Not_Recruiting	NCT01204684	2010
	Early_Phase1	Recurrent Melanoma	Completed	NCT01748747	2012
	Phase1 and 2	Melanoma	Unknown	NCT02126579	2014
	Phase4	Postoperative Pain	Completed	NCT03570541	2018
	Phase1 and 2	Advanced Solid Tumour	Recruiting	NCT04799054	2021
	Phase1 and 2	Non-muscle-invasive Bladder Cancer	Recruiting	NCT05710848	2023
CpG ODN	Phase2	Lymphoma, Mantle-Cell	Completed	NCT00490529	2007
	Early_Phase1	Breast Cancer	Completed	NCT00640861	2008
	Phase2	Breast Cancer	Terminated	NCT00824733	2009
	Phase1	Melanoma	Completed	NCT01149343	2010
	Phase2	Malignant Melanoma	Recruiting	NCT04126876	2019
	Phase1	Pancreatic Cancer and Metastatic Pancreatic Cancer	Recruiting	NCT04612530	2020
	Phase1	Lung Cancer and Hepatocellular Carcinoma and Solid Tumour	Recruiting	NCT04952272	2021
Poly(I:C)	Phase1	Prostate Cancer	Completed	NCT03412786	2018
	Phase1	Leiomyosarcoma	Active_Not_Recruiting	NCT04420975	2020
	Early_Phase1	Advanced Hepatocellular Carcinoma	Terminated	NCT04777708	2021
CMP-001	Phase1 and 2	Advanced Cancer	Terminated	NCT02554812	2015
	Phase1	Non-Small Cell Lung Cancer	Completed	NCT03438318	2018
	Phase1	Colorectal Neoplasms Malignant and Liver Metastases	Completed	NCT03507699	2018
	Phase2	Melanoma and Lymph Node Cancer	Active_Not_Recruiting	NCT03618641	2018
	Phase1 and 2	Lymphoma	Recruiting	NCT03983668	2019
	Phase1 and 2	Locally Advanced Malignant Solid Neoplasm	Terminated	NCT04387071	2020
	Phase2	Melanoma	Recruiting	NCT04401995	2020
	Phase2	Squamous Cell Carcinoma of Head and Neck	Active_Not_Recruiting	NCT04633278	2020
	Phase2	Triple Negative Breast Cancer	Recruiting	NCT04807192	2021
	Phase2	Merkel Cell Carcinoma, Triple Negative Breast Cancer and Non-Small Cell Lung Cancer	Recruiting	NCT04916002	2021
	Phase3	Solid Tumours	Recruiting	NCT05059522	2021
	Phase2	Multiple Primary Cancers	Not_Yet_Recruiting	NCT05164510	2021
	Phase2	Metastatic Prostate Adenocarcinoma	Not_Yet_Recruiting	NCT05445609	2022
TREM2 inhibitor					
PY314	Phase1	Advanced Solid Tumour	Recruiting	NCT04691375	2020
Clever 1 inhibitor					
FP-1305	Phase1 and 2	Cancer	Recruiting	NCT03733990	2018
	Phase1	Non-small Cell Lung Cancer	Not_Yet_Recruiting	NCT05171062	2021
	Phase1 and 2	Acute Myeloid Leukemia	Recruiting	NCT05428969	2022
Complement inhibitor					
IPH5401	Phase1	Advanced Solid Tumours	Terminated	NCT03665129	2018
Macrophage cell therapy					
CT-0508	Phase1	Solid Tumours		NCT04660929	2020
TEMFERON	Phase1 and 2	Glioblastoma Multiforme	Recruiting	NCT03866109	2019
	Phase1 and 2	Multiple Myeloma	Terminated	NCT03875495	2019

### Depletion of macrophages

TAM recruitment by CCL2 and CCR2 is critical to tumour invasion and metastasis (Xu et al., 2021b). CCL2-CCR2 signaling controls the supply of circulating inflammatory monocytes (Argyle and Kitamura, 2018) and inhibiting CCR2 keeps monocytes in bone marrow, reducing TAMs at cancer sites (Flores-Toro et al., 2020). Blocking CCL2-CCR2 axis also hinders TAM recruitment, decreasing tumour incidence and enhancing CD8^+^ T cells anti-tumour activity (Teng et al., 2017; Tu et al., 2020). Another target is CSF-1, which promotes monocyte and macrophage differentiation, proliferation, and function (Stanley and Chitu, 2014). Mouse models with CSF-1R inhibition had smaller tumors and better survival (Tan et al., 2021). Small molecule inhibitors of CSF1-R have also been shown to deplete some TAMs, enhancing tumour sensitivity to chemotherapy (O'Brien et al., 2021).

### Alteration of macrophage phenotypes

TAMs change into a tumour-suppressing phenotype (Liu et al., 2021) which is a promising clinical strategy for cancer treatment. Inducing M1 macrophage phenotype through the use of selective class IIa HDAC inhibitors (Li et al., 2021a) enhances T cell responses to chemotherapy and immune checkpoint blockades (McCaw et al., 2019). The CD47/SIRP-α pathway is crucial for tumour immune escape, and blocking it enhances macrophages immune killing against tumours (Wang et al., 2020; Jia et al., 2021). Cancer immunotherapy research has also focused on anti-PD-1/PD-L1 treatment (Tomlins et al., 2023). TAMs, particularly M2 TAMs, express PD-L1 on their surface and contribute to immunosuppression by promoting T-cell apoptosis (Li et al., 2022b; Shinchi et al., 2022). In vitro-transcribed mRNA could stimulate effector molecule synthesis or cell reprogramming. mRNA in an injectable nanocarrier genetically reprogrammed TAMs into antitumour effectors. Nanoparticles formulated with mRNAs encoding the transcription factor interferon regulatory factor 5 (IRF5) and its activating kinase, inhibitor of NF-B kinase subunit-β (IKKβ), reversed the immunosuppressive TME and reprogrammed TAMs, regressing tumours in mouse cancer models (Zhang et al., 2019; Petty et al., 2021). The LILRB family, specifically LILRB2, is integral to the immune evasion strategies of cancer cells (Chen et al., 2018). LILRB2, an MHC-binding protein rich in TAMs, interacts with MHC class I molecules, which cancer cells often downregulate to dodge T cell recognition (Liu et al., 2023c). Blocking LILRB2 enhances macrophage pro-inflammatory and phagocytic activity. Its effect on macrophage activation and phagocytosis is unknown (Chen et al., 2018). MK-4830, an antibody against LILRB2, showed promising results in early trials with advanced-stage tumours (Siu et al., 2022). Responses correlated with the expression of pro-inflammatory cytokines and enhanced cytotoxic T cell-mediated anti-tumour immune response (Sharma et al., 2021). These approaches have been tested with other clinical used immunotherapies like immune checkpoints for their clinical potential with animal models and clinical trials.

### Antigen presentation enhancement

Scavenger receptors on TAMs are becoming therapeutic targets for their role in promoting TME pro-inflammatory shifts. Scavenger receptor CD163 is associated to tumour progression in several malignancies but the mechanism is unclear (Xie et al., 2022). However, CD163+ macrophage depletion causes tumor regression and re-establish anti-PD1 treatment response (Etzerodt et al., 2019). Macrophage mannose receptor 1 (MRC1), also known as CD206, affects tumour immunity (Rahabi et al., 2020). Its activation induces immunosuppressive macrophages. Intriguingly, MRC1-binding peptide RP-182 converts TAMs into anti-tumour M1-like effector cells (Jaynes et al., 2020). The collagenous macrophage receptor (MARCO) is abundantly present on TAMs. Targeting MARCO potentially reprogrammes TAMs from tumour-supportive to pro-inflammatory effectors (Sa et al., 2020; La Fleur et al., 2021). Another scavenger receptor Clever 1 also suppresses macrophages and T helper 1 lymphocytes (Virtakoivu et al., 2021). Blocking it switches TAMs from immunosuppressive to pro-inflammatory (Viitala et al., 2019). Triggering receptor expressed on myeloid cells 2 (TREM2), upregulated on TAMs in human and mouse tumours, is a potential target (Katzenelenbogen et al., 2020; Molgora et al., 2020). Blocking TREM2+ macrophages limit tumour growth and augment anti-PD1 therapy (Binnewies et al., 2021). PSGL1, highly expressed in TAMs, represents a valuable target for TAMs re-education (Johnston et al., 2019). Using anti-PSGL1 monoclonal antibody potentially triggers a pro-inflammatory response in tumour tissues, exhibiting notable antitumour activity (DeRogatis et al., 2022; Lin et al., 2023).

### Innovative strategies for TAM modulation

Recent strategies explore TAM modulation. One approach involves the engineering of T cells with chimeric antigen receptors (CAR) (Maalej et al., 2023) specifically tailored to recognize and eliminate TAMs. Research shows CAR T cells targeting macrophages are effective against various solid organ tumours, including ovarian and pancreatic cancer (Sanchez-Paulete et al., 2022). Eliminating M2-like FRβ+ TAMs in the murine models of ovarian cancer, colon cancer and melanoma TME through FR-specific CAR-T cells delay tumour progression and prolong life (Rodriguez-Garcia et al., 2021). These CAR-engineered T cells show potential in redirecting immune responses against the tumour. Another method focuses on harnessing invariant natural killer T (iNKT) cells (Li et al., 2021b). These cells possess innate and adaptive immune properties, CAR-iNKT cells use iNKT TCR/CD1d and CAR recognition to deplete TAMs and tumours (Simonetta et al., 2021). Recent studies harness iNKT cells to modulate TAMs, boosting antitumour responses. Other innate T cells, including MAIT, and γδT cells, have potential clinical applications as they target and eliminate TAMs (Li et al., 2022c). In synthesis, these innovative strategies signify a shift in tumour immunotherapy (Table 2).

**TABLE 2:** Innovative strategies targeting TAMs in tumour microenvironment.

Cell type	Tumour type	Function
FRβ.CAR-T	Ovarian, Pancreatic, Colon, Melanoma	Recognize and eliminate TAMs, delay tumour progression and prolong life
F4.CAR-T	Orthotopic Lung Tumours	Deplete TAMs, inhibit tumour growth, enhance MHC upregulation via IFNγ, and boost CD8 T cell expansion and tumour cell immune editing
iNKT	Melanoma, Multiple myeloma, Ovarian	Use iNKT TCR/CD1d and CAR recognition to deplete TAMs
γδT		Raise MDSCs, induce antitumour responses with zoledronic acid, target monocytes, and kill macrophages
MCAR-MAIT		Kill OVCAR3-FG tumour cells, have dual CAR/TCR targeting mechanisms, sustain antitumour capacity in presence of macrophages, and target TAMs

## Prospects of macrophages in cancer

TAMs are an important immune cell type that shapes TME properties. Targeting TAMs effectively blocks the progression of various cancer types. Moreover, popularity of single-cell RNA-sequencing analysis enhances the mechanistic study and preclinical research of TAMs in TME (Tang et al., 2020; Tang et al., 2021a; Chung et al., 2023). Dissecting the heterogeneity and regulatory mechanism of macrophages in cancer at single-cell resolution leads to the discovery of novel macrophage-specific therapeutics targets from the TME, for example, MMT and MNT (Xue et al., 2021; Tang et al., 2022a; Tang et al., 2022b). They are emphasizing the adaptive plasticity of macrophages. MMTs, derived from M2 TAMs with protumour activities, lead to the formation of CAFs. These CAFs are key in driving cancer progression (Chen and Song, 2019; Li et al., 2020). The roles of MMT-derived CAFs in functions, including adaptive immunity suppression, drug resistance, metastasis, and promoting cancer cell stemness warrant investigation. Conversely, MNTs highlight the transformation of TAMs into neuron-like entities, influencing *de novo* neurogenesis in the TME (Tang et al., 2022b) and contributing to cancer-associated pain (Shepherd et al., 2018). This transition, while prevalent in NSCLC, is also seen in other tumours, emphasizing its importance in cancer pain and tumour innervation (Tang et al., 2022b). Given the impact of cancer pain on quality of life, especially in patients with advanced stages of the disease (Wang et al., 2021c), understanding MNT is vital for pain management strategies. Notably, these transitions were found to be mediated by a Smad3-centric gene network in TAMs, highlighting the potential of macrophage-targeted Smad3 interventions as a promising therapeutic approach in cancer immunotherapy (Tang et al., 2017; Feng et al., 2018; Tang et al., 2021b; Tang et al., 2022b). These new findings lead to the development of effective therapeutic approaches to enhance the efficiency of conventional anticancer treatments as well as the latest immunotherapies which are not primary or secondary resistant in patients with solid cancers (Kim et al., 2019b; Kim et al., 2020; Tang et al., 2020; Chung et al., 2021; Xue et al., 2021). Besides, macrophages are considered as a primary target of anti-inflammatory therapy for cancer prevention, their therapeutic potential is explored by new trials worldwide (Tang et al., 2019; Lee et al., 2021; Tang et al., 2022d). Despite the challenges, a better understanding of the immunodynamics of TAM shows a substantial potential for improving the therapeutic efficiency and clinical outcomes of cancer patients in the future.
